# pRR30, pRR3.25% and Asymmetrical Entropy Descriptors in Atrial Fibrillation Detection

**DOI:** 10.3390/e26040296

**Published:** 2024-03-28

**Authors:** Bartosz Biczuk, Szymon Buś, Sebastian Żurek, Jarosław Piskorski, Przemysław Guzik

**Affiliations:** 1 Institute of Physics, University of Zielona Góra, 65-069 Zielona Góra, Poland; s.zurek@if.uz.zgora.pl (S.Ż.); jaropis@zg.home.pl (J.P.); 2 The Doctoral School of Exact and Technical Sciences, University of Zielona Góra, 65-417 Zielona Góra, Poland; 3 Institute of Electronic Systems, Faculty of Electronics and Information Technology, Warsaw University of Technology, 00-650 Warszawa, Poland; szymon.bus.dokt@pw.edu.pl; 4 Department of Cardiology—Intensive Therapy, Poznan University of Medical Sciences, 60-355 Poznań, Poland; pguzik@ptkardio.pl; 5 University Centre for Sports and Medical Studies, Poznan University of Medical Sciences, 60-802 Poznań, Poland

**Keywords:** atrial fibrillation, cardiac arrhythmia, electrocardiography, heart rate variability, entropy

## Abstract

Background: Early detection of atrial fibrillation (AF) is essential to prevent stroke and other cardiac and embolic complications. We compared the diagnostic properties for AF detection of the percentage of successive RR interval differences greater than or equal to 30 ms or 3.25% of the previous RR interval (pRR30 and pRR3.25%, respectively), and asymmetric entropy descriptors of RR intervals. Previously, both pRR30 and pRR3.25% outperformed many other heart rate variability (HRV) parameters in distinguishing AF from sinus rhythm (SR) in 60 s electrocardiograms (ECGs). Methods: The 60 s segments with RR intervals were extracted from the publicly available Physionet Long-Term Atrial Fibrillation Database (84 recording, 24 h Holter ECG). There were 31,753 60 s segments of AF and 32,073 60 s segments of SR. The diagnostic properties of all parameters were analysed with receiver operator curve analysis, a confusion matrix and logistic regression. The best model with pRR30, pRR3.25% and total entropic features (H) had the largest area under the curve (AUC)—0.98 compared to 0.959 for pRR30—and 0.972 for pRR3.25%. However, the differences in AUC between pRR30 and pRR3.25% alone and the combined model were negligible from a practical point of view. Moreover, combining pRR30 and pRR3.25% with H significantly increased the number of false-negative cases by more than threefold. Conclusions: Asymmetric entropy has some potential in differentiating AF from SR in the 60 s RR interval time series, but the addition of these parameters does not seem to make a relevant difference compared to pRR30 and especially pRR3.25%.

## 1. Introduction

Atrial fibrillation (AF) is a cardiac arrhythmia with irregular heartbeats. It can lead to the development of tachyarrhythmias, heart failure, dementia and arterial emboli, with ischaemic cerebral stroke being the most common complication [1]. AF also increases the risk of dying prematurely.

AF is often asymptomatic, especially in men and older people [2], and its complications, e.g., ischaemic stroke, may be the first presentation of this arrhythmia. The incidence of AF increases with age and is usually associated with many diseases or risk factors like hypertension, obesity, smoking, coronary artery disease, valvular heart disease, lung disease and hyperthyroidism [3]. However, AF is not rare in otherwise healthy people, e.g., those involved in long-term endurance sports [4]. AF can also be caused by alcohol consumption and the abuse of illicit substances such as methamphetamine, cocaine, opiates and cannabis [5].

An increasing incidence of AF and its clinical importance require effective and timely diagnosis. Recently, parameters derived from heart rate variability (HRV) analysis have become more commonly applied for AF detection in ECGs [6]. Several HRV descriptors have been proposed, such as the standard deviation of the interbeat intervals (SDRR), root mean square of successive differences between normal heartbeats (rMSSD) and percentage of differences higher than 50 ms (pRR50) [7].

pRR50 is the most studied but specific example of a parameter from the pRRx family (percentage of successive RR interval differences greater than or equal to x ms). Buś et al. [8] explored different parameters from the pRRx family and found that the optimal diagnostic properties for AF detection were found for the threshold x = 31 ms (AUC = 0.958, sensitivity = 95.35%, specificity = 90.47). The study was performed on a dataset of over sixty thousand 1 min segments of RR intervals. Using the same data, they reported that the pRRx% parameters (defined like pRRx but with a threshold x% relative to the previous RR interval) have even better diagnostic properties with pRR3.25% (AUC = 0.972, sensitivity = 97.16%, specificity = 93.75%) outperforming other pRRx% and pRRx parameters. Both the pRRx and pRRx% families count occurrences of unusually large successive differences in RR intervals and are generally much greater in AF than in normal sinus rhythm (SR).

Asymmetric entropy based on monotonic runs, introduced by Piskorski and Guzik [9], is another set of features used in HRV analysis. Unlike other entropy measures, asymmetric entropy separately examines information derived from monotonic runs consisting of heart rate accelerations, decelerations or consecutive RR intervals that do not change (neutral monotonic runs).

We hypothesized that heart rate entropy derived from deceleration, acceleration and neutral runs might differ between ECGs derived from sinus rhythm and atrial fibrillation. If so, the measurement of asymmetric entropy may be useful in distinguishing SR and AF segments of the ECG. This study compared the asymmetric entropy-based descriptors with pRR30 and pRR3.25% and their possible combinations for AF detection in 1 min segments of RR intervals.

## 2. Materials and Methods

### 2.1. Data

This study used de-identified data from the Long-Term Atrial Fibrillation Database (LTAFDB) [10,11]. The LTAFDB consists of 84 extended 24 h Holter electrocardiographic (ECG) recordings sampled at 128 Hz. The database contains information on R-wave locations and corresponding heart rhythms (normal, supraventricular, ventricular, atrial and technical artefacts) in patients with paroxysmal AF and other arrhythmias. For our analysis, we selected only continuous ECG fragments with either AF or sinus rhythm (SR) lasting at least 60 s, discarding segments labelled as other rhythms.

**Figure 1:**
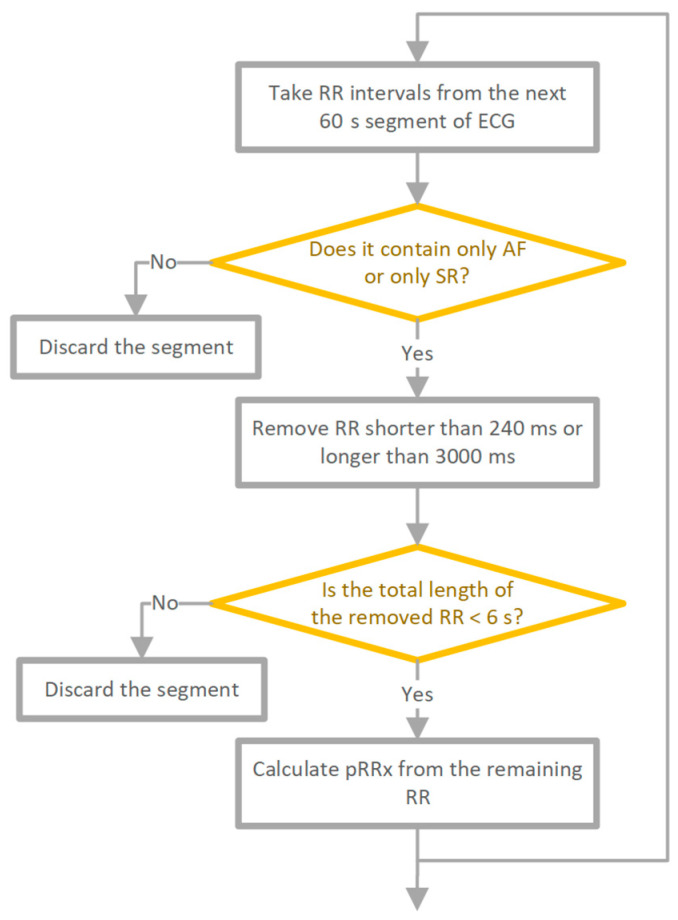
Data pre-processing scheme [8].

The data pre-processing method used is shown in Figure 1. The RR interval time series were divided into 60 s contiguous segments. Each RR interval within the segment had to originate from SR to label the whole segment as SR. If not, the segment was excluded from further analysis. Similarly, for AF segments, each beat within the segment had to be AF, and segments containing ventricular beats were also excluded. To minimize the number of potentially unidentified technical artefacts, we removed RR intervals shorter than 240 ms or longer than 3000 ms for both SR and AF. Segments where the total length of excluded RR intervals exceeded 3% (1.8 s) of the segment length were excluded from the analysis. The choice of a 3% threshold for excluding segments based on the total length of excluded RR intervals was influenced by established guidelines in the field. Specifically, [1] advocate for the exclusion of segments where more than 10% of RR intervals are not of sinus origin or are artifacts. Our decision to narrow this threshold to 3% was primarily due to the specific context of our study, which involved very short 1 min segments. After pre-processing, the total 60 s RR series was 63,636 (31,919 SR, 31,717 AF). ECG segments where the total length of RR intervals after filtering was less than 58 s were also discarded.

### 2.2. Software

We utilized the Python programming language (version 3.9, Python Software Foundation, Wilmington, DE, USA) for statistical analysis. Specifically, for matrix manipulation, we employed the NumPy library, while plots were generated using the Matplotlib library. For training and testing logistic regression models, we utilized the Scikit-learn library.

In our analysis, part of the runs-based asymmetrical calculation was facilitated by the HRAexplorer v0.1.0, a free GPL3 software written in Python, available for review and download at https://github.com/jaropis/pyHRAExplorer, accessed on 26 March 2024. HRAexplorer is an open-source online tool tailored for HRV and heart rate asymmetry (HRA) analysis. It enables the calculation of various HRV and HRA descriptors, as well as the statistical and visual analysis of uploaded RR intervals time series. An interactive online version of this software in the R programming language may be found at https://hraexplorer.com/ and source code can be found at https://github.com/jaropis/HRAExplorer, (accessed on 26 March 2024).

Additionally, data pre-processing and the calculation of pRRx and pRRx% parameters were conducted using code sourced from https://github.com/simonbus/prrx_af (accessed on 26 March 2024). This repository contains Python scripts designed for pre-processing data from the Long-Term Atrial Fibrillation Database LTAFDB, including data extraction, RR intervals annotation and filtering.

### 2.3. Asymmetrical Entropy

**Figure 2:**
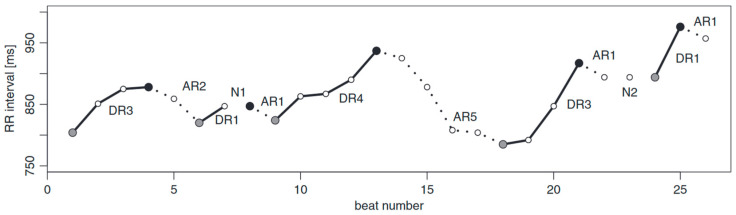
Example of runs-based partitioning of a short tachogram. The runs can be classified as deceleration and acceleration runs of length i (DRi and ARi, respectively), with neutral runs represented by the symbol Ni, which can interrupt the deceleration/acceleration runs. A full grey circle indicates the start of a deceleration run. A full black circle marks the start of an acceleration run. These circles can be used as reference points for the respective runs [9].

Asymmetrical entropy is an approach presented in [9]. In summary, the RR series is partitioned into monotonic runs, as shown in Figure 2. If we define a series of RR intervals as follows:

$${RR}_{N}\equiv ({RR}_{1},{RR}_{2},\ldots ,{RR}_{N})$$

We can use this to build a series of differences. Starting from the second element, we subtract the (*i* − 1)th element from the *i*-th element as follows:

$$\Delta \equiv (\delta_{1},\delta_{2},\ldots ,\delta_{N-1})$$

For the above equation, the single difference looks like the following:

$$\delta_{i}={RR}_{i+1}-{RR}_{i}$$

We can use this series to construct a symbolic series of signs of those differences:

$$sgn\left(\delta_{i}\right)=\{+,\;{\;\delta }_{i}>0-,\;{\;\delta }_{i}<0\;0,\;{\;\delta }_{i}=0$$

Using this, we can write definitions for the acceleration, deceleration and neutral series:

**Definition 1.**  *A deceleration series of length i (DRi) is an uninterrupted series of i decelerations (sign +) that starts and ends with an acceleration (sign −) or neutral difference (sign 0).*

**Definition 2.**  *An acceleration series of length i (ARi) is an uninterrupted series of i accelerations (sign −) that starts and ends with a deceleration (sign +) or neutral difference (sign 0).*

**Definition 3.**  *A neutral series of length i (NRi) is an uninterrupted series of i neutral differences (sign 0) that starts and ends with an acceleration (sign −) or deceleration (sign +).*

It is common practice [9,10,11] to remove neutral runs from the record by adding white noise with a sufficiently small standard deviation. However, we will include neutral runs in our calculation and model building.

We calculate the Shannon entropy for each type of the monotonic run. If we define the value *p* as a probability estimate of how likely it is to find an interval in the RR recording that belongs to a given run type of a given length:

$$p_{i,k}=\frac{\left(number\;of\;r_{i}^{k}\right)\times i}{n}$$

where *i* is the run length and *k* is the run type, which can be an AR, DR or NR, the Shannon entropy for this estimator is as follows:

$$H_{k}=-\sum_{i=1}^{{max(i)}_{k}}p_{i,k}\cdot ln{\;p}_{i,k}$$

Mathematical details can be found in [9,12,13].

### 2.4. pRR30 and pRR3.25%

The pRR50 metric is a well-known but singular instance within the broader pRRx category, which quantifies the percentage of consecutive RR interval variances exceeding x milliseconds. In their comprehensive study, Buś et al. (2022) [8] conducted an extensive examination of various pRRx family parameters. They identified that the threshold of 31 ms yielded the most favourable diagnostic characteristics for identifying atrial fibrillation (AF), demonstrating an area under the curve (AUC) of 0.958, with a sensitivity of 95.35% and a specificity of 90.47%. This analysis was based on an expansive collection of over 60,000 one-minute RR interval segments.

Further analyses in [8] revealed superior diagnostic capabilities within the pRRx% subgroup, which is akin to pRRx but applies a percentage threshold relative to the preceding RR interval. Specifically, the parameter pRR3.25% emerged as notably effective, surpassing other pRRx and pRRx% metrics. This parameter achieved an AUC of 0.972, a sensitivity rate of 97.16% and a specificity of 93.75%, indicating a significant improvement in detecting AF compared to traditional methods. Both pRRx and pRRx% parameters function by detecting significant successive variations in RR intervals, which are markedly more prevalent in cases of AF compared to normal sinus rhythm [8]. For these reasons we have decided to make pRR3.25% and pRR30, rather than pRR50, the focus of this study.

### 2.5. Statistical Analysis

To establish the normality of the data we used the Shapiro–Wilk normality test [14] and confirmed the numerical results from this test by visually inspecting the qq plot of the data. Non-parametric Spearman correlation was used to analyse associations between parameters. The receiver operating characteristic (ROC) [15] was used to investigate which parameters discriminated between the 60 s segments of sinus rhythm (SR) and atrial fibrillation (AF). For each HRV parameter studied, Youden’s criterion [16] was used to calculate the optimal cut-off from the ROC curve [17].

We used a 2:1 ratio to divide the final set of 63,826 recordings into the training dataset (42,763 samples) and the test dataset (21,063 samples). The main reason for selecting this ratio was striving for a balanced trade-off [18]. Given the relatively low complexity of our models (e.g., the number of parameters), and the large dataset, we felt that giving ⅓ of the data to the test set would not impair the training process, while at the same time giving us more certainty that our results are not the effect of randomness. This makes our approach more conservative and less prone to accidental findings. Univariate and multivariate logistic regression examined individual parameters or their combinations with either pRR30 or pRR3.25% or both pRR30 and pRR3.25%. A non-parametric bootstrap with 1000 samples was used to estimate classification metrics with a 95% confidence interval. Only *p* < 0.05 was considered statistically significant.

## 3. Results

Our data consisted of 64,738 segments, from which 912 were discarded after filtration, leaving 63,826 segments. A total of 31,753 of them were classified as AF and 32,073 as SR. The mean RR was 698 ms for AF and 858 ms for SR.

### 3.1. Entropy Distribution

**Figure 3:**
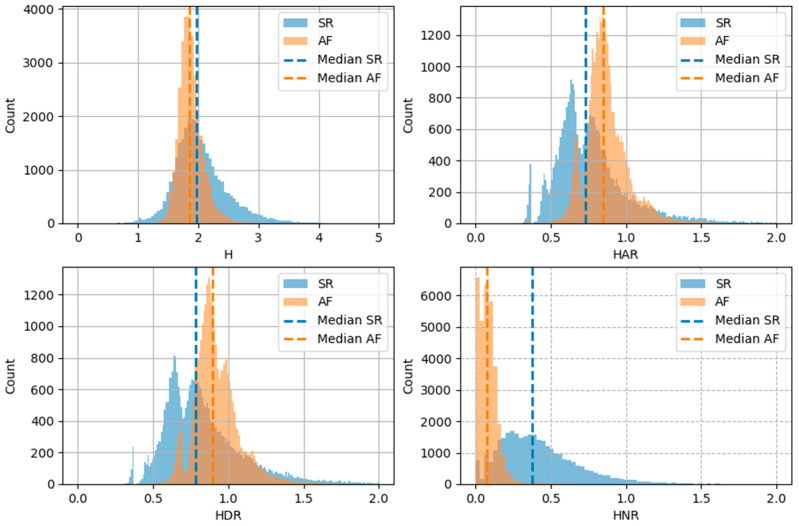
Histograms of different types of asymmetrical entropy for AF and SR.

Figure 3 presents histograms for all studied types of asymmetrical entropy: the total entropy (H), entropy of acceleration runs (HAR), entropy of deceleration runs (HDR) and entropy of neutral runs (HNR). All forms of entropy have narrower and higher histograms for AF than for SR. The peaks of the histograms for H overlap for AF and SR, but are separated for HAR, HDR and HNR. The median HAR and HDR were higher for AF than for SR. In contrast, the median H and HNR were lower in AF.

**Figure 4:**
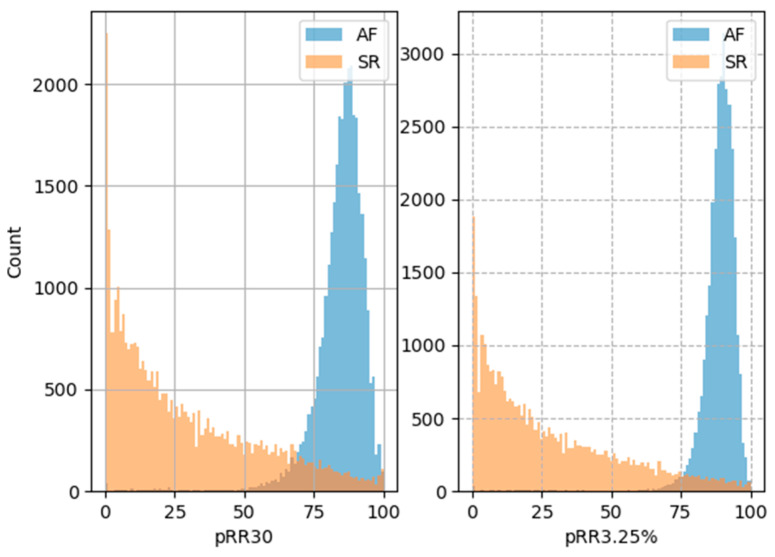
Histograms of prr30 and prr3.25% AF and SR recordings.

Figure 4 shows how the histograms of the pRR30 and pRR3.25% differ in AF and SR. For SR, the most common values of pRR30 and pRR3.25% were close to 0%. The rates gradually decreased for higher values of pRR30 and pRR3.25%. In contrast, the distributions are shifted to the right for AF and the most common values of pRR30 and pRR3.25% were around 85% and 90%, respectively.

**Figure 5:**
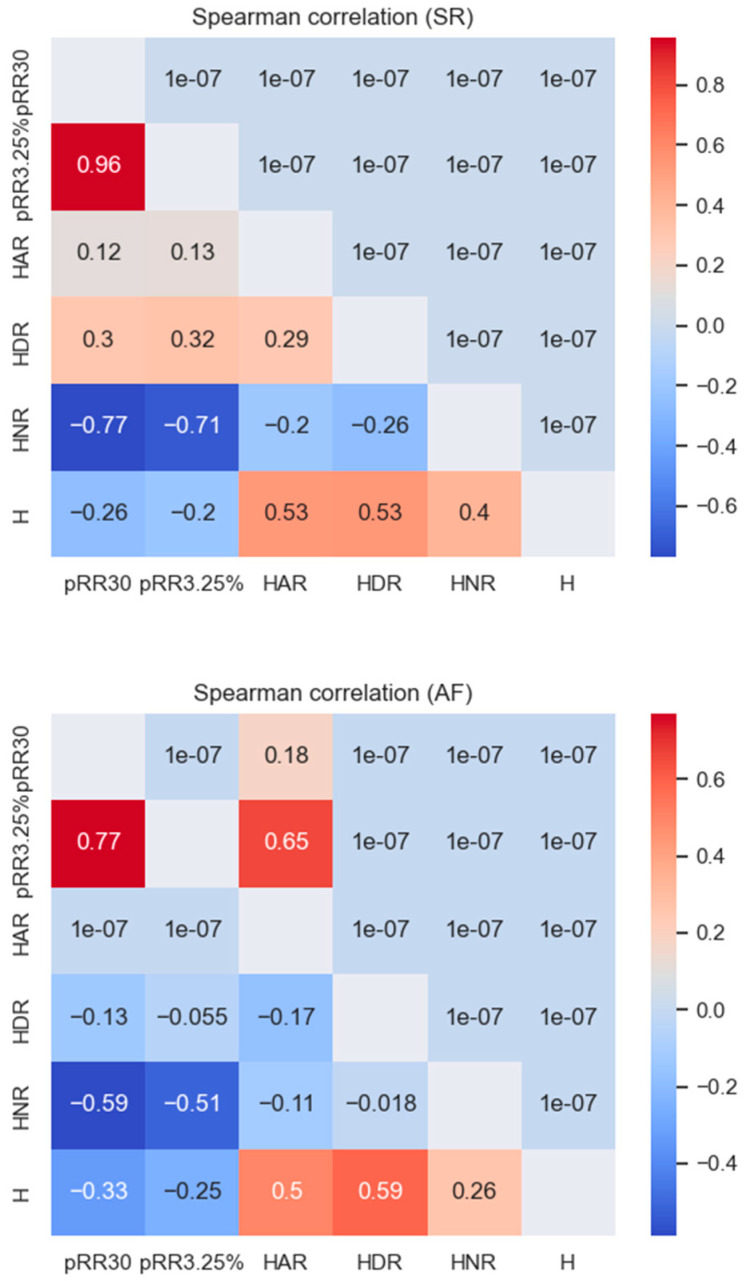
Spearman correlation matrix for HRV parameters for SR and AF data. Lower triangle: correlation coefficients; upper triangle: *p*-values.

Figure 5 shows the Spearman correlation values between all of the features examined for the SR and AF data separately. For SR, HNR correlated strongly and negatively (dark blue) with pRR30 (−0.77) and pRR3.25% (−0.71). The correlations between HNR and pRR30 and pRR3.25% were weaker for AF, with the most pronounced correlations for HNR and pRR30 (−0.59).

### 3.2. Diagnostic Properties of Single HRV Parameters

Table 1 characterizes the diagnostic properties of all investigated parameters to differentiate AF from SR for the 1 min ECG segments. As pRR3.25% has the highest AUC, it is considered the reference value for comparisons with other parameters and is included in all multivariate diagnostic models. AUC for the discrimination of AF from SR for the 1 min RR interval ECG segments were all significantly different from 0.5. (*p* at least < 1 × 10^−114^). The top three strongest candidates for AF detection are pRR3.25% (AUC = 0.9727) pRR30 (0.9596) and HNR (0.9315).

**Figure 6:**
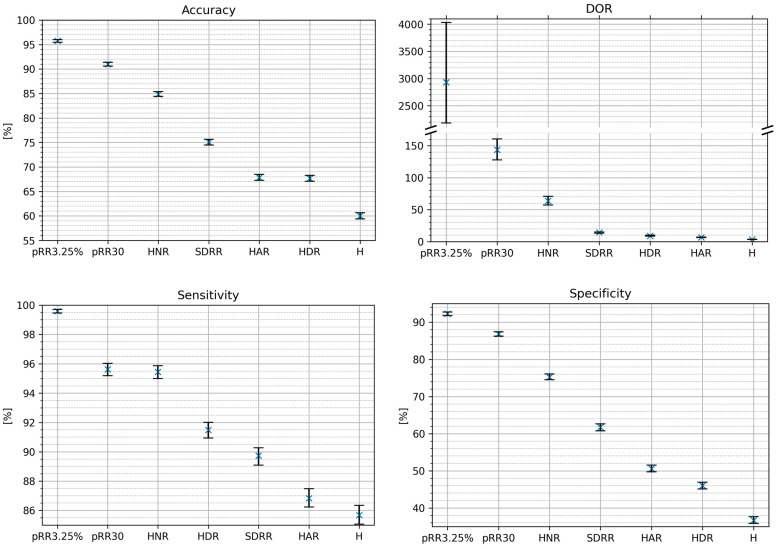

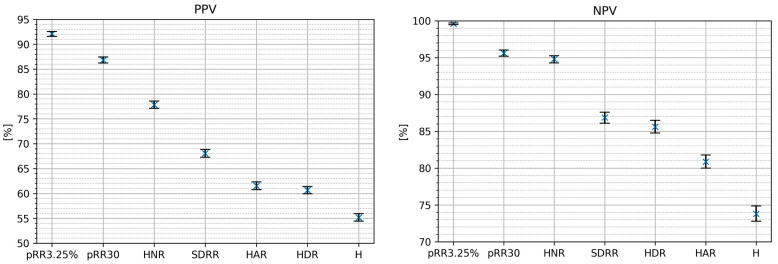
Classification metrics of AF detection on the test dataset using the optimal cut-off method. DOR—diagnostic odds ratio [17], PPV—positive predictive value, NPV—negative predictive value.

Figure 6 shows the classification metrics for AF detection on the testing dataset using the optimal cut-off method for single HRV parameters. For every metric, the best score achieved three parameters: pRR3.25%, pRR30 and HNR. The NPV difference between pRR30 and HNR was the smallest. The whiskers on the plots represent 95% confidence intervals from the bootstrap.

### 3.3. Diagnostic Values of Models Built Using pRR30, pRR3.25% and Asymmetric Entropy Indices

Table 2 presents results of AF detection using univariate or multivariate logistic regression models on the testing dataset. The highest AUC was achieved by the model with three parameters, pRR30, pRR3.25% and H, while pRR30, pRR3.25% and HAR had the highest DOR. From the univariate models, pRR3.25% had the highest AUC and all other metrics.

**Figure 7:**
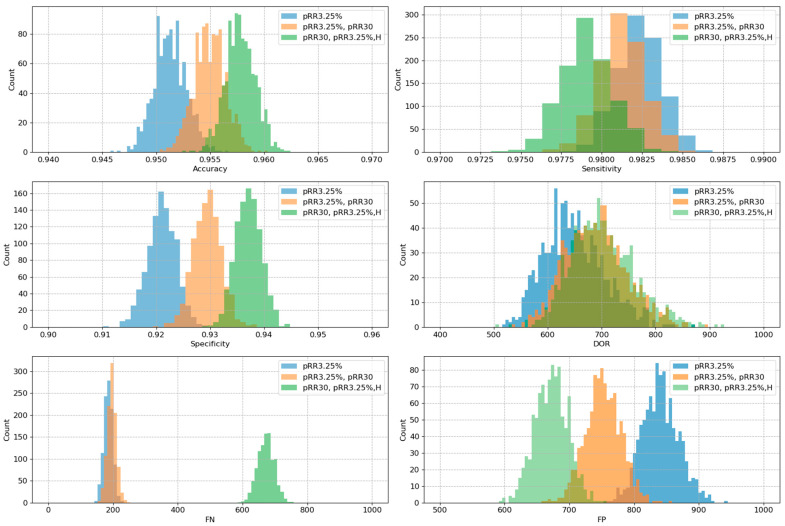
Histograms presenting the 1000-times bootstrapped results of the model built from pRR3.25% vs. pRR30 and pRR3.25% vs. the model built from pRR30, pRR3.25% and HAR. The biggest difference was in FNs where adding H to the model increases the number of FNs over three times compared to models without H.

From Table 2 we selected three models for further validation. These included the best (in AUC) single-feature model, which was pRR3.25%, the best two-feature model, which was pRR3.25% and pRR30 and the best three-feature model which was pRR3.25%, pRR30 and H. A 1000-resampled bootstrap test revealed a statistically significant difference in AUC (*p*-value = 0.02) between the one-feature and two-feature models. Furthermore, the comparisons between the one-feature versus three-feature and two-feature versus three-feature models yielded p-values less than 10^−6^, indicating highly significant differences. The same bootstrap methodology was employed to assess the performance of the models in terms of accuracy, sensitivity, specificity, DOR, FNs and FPs. In all cases, the differences between the models were statistically significant, with *p*-values consistently below 10^−5^. Figure 7 provides histograms with a visual representation of these findings.

It is worth noting that the scales in Figure 7 for accuracy, sensitivity and specificity on the respective x-axes have been adapted. In this way it is easier to see the small differences. In practice, however, the range of these differences is negligible. Optimizing the models for AUC occurs at the expense of not detecting AF. The median of FNs for pRR3.25% alone and for pRR3.25% with pRR30 increased from less than 200 to almost 700 undetected AF for the model with pRR3.25%, pRR30 and H.

## 4. Discussion

We found that both pRR3.25% and pRR30 outperformed all asymmetric entropy parameters in their diagnostic value for detecting AF in 1 min ECGs. For AF detection, the best single parameter was pRR3.25% with an AUC of 0.972 and a DOR of 643, the second best was pRR30 with an AUC of 0.959 and a DOR of 233.5, and the third best was HNR with an AUC of 0.93 and a DOR of 84.5. All combinations that had either a higher AUC or DOR than a single pRR3.25% contained pRR3.25%. The best combination when measured by AUC was pRR3.25%, pRR30 and H, while when measured by DOR it was pRR3.25% and H. However, the combined models with higher AUC or DOR had increased false negative values. Using pRR3.25% as a reference, the observed increases in AUC were of negligible clinical value as they were up to 0.006, which is a less than 1% improvement. In addition, using the best combination, i.e., pRR3.25%, pRR30 and H, was associated with a risk of increasing false-negative AF diagnoses—the number of undiagnosed AF episodes increased almost fourfold.

This study is not the first one to demonstrate entropy-based features for AF detection. In [19] Richman JS and Moorman JR have introduced sample entropy, a novel method for comparing two physiological time series. Liu C et al. and Zhao L et al. [20,21] have compared different entropic measures and proposed a novel one, called normalized fuzzy entropy for AF detection. They have achieved 0.86 accuracy for 60 beat-long RR segments. Their approach and other tested features were based on sample entropy [19].

Runs-based asymmetrical entropy is very different from sample entropy. It does not depend on parameters that represent matching radius for determining similarity between sequences of data points within a time series and can break relative consistency, which means that it does not always provide consistent results when the sample size increases [22]. Run-based asymmetric entropy has a more straightforward interpretation. It quantitatively separates the contributions of accelerating, decelerating and neutral runs to the overall asymmetry of the time series (Figure 2).

We found a negative correlation between the asymmetric entropy of NR and pRR30. In addition, the HNR distribution differed between AF and SR. The AF classifier built from HNR alone had an accuracy of over 0.88, the best among other run-based asymmetric entropy parameters. Neutral runs are either ignored, removed from the analysis or replaced by the addition of white noise [9,12,13]. However, neutral runs may contain valuable information, especially in the context of AF detection. Typically, their contribution to HRV is either zero (to short-term HRV) or random and depends on the ECG sampling frequency. However, their lower rate in AF provides interesting information.

The nature of AF makes RR intervals highly random, with low stability. This results in a low probability of finding two or more consecutive RR intervals of the same duration. It also explains why there are fewer neutral runs in AF than in SR. Changes in RR interval duration in SR are gradual. They are controlled by many physiological mechanisms that modulate sinus node depolarisation. In AF, fast fibrillation waves above 350 depolarisations/minute reset and quieten the sinus node. Changes between successive RR intervals in AF are less well controlled and therefore more dramatic. We have previously reported higher values of pRRx and pRRx% in AF than in SR [8]. Therefore, the probability of small changes between consecutive RR intervals is low in AF but higher in SR. Here we show that information derived from neutral runs has some diagnostic properties. However, it is uncertain whether this information should be incorporated into AF detection algorithms. All run-based asymmetric entropy measures, either as single parameters or in combination with pRR3.25% or pRR30, are not particularly effective in discriminating AF from SR. They are also more complex to calculate than pRR3.25% or pRR30.

As previously shown, the pRRx and pRRx% families have interesting diagnostic properties for distinguishing ECG segments with AF from SR. First, we demonstrated that pRR30 outperformed other commonly used HRV indices including SDNN, SD1, SD2, SD2/SD1 and the coefficient of variation, for the same purpose [8]. Second, we showed that pRR30 is outperformed by the pRRx% family, especially pRR3.25%. In this study, we report that both pRR30 and pRR3.25 have better diagnostic properties for AF detection than any of the run-based asymmetric entropy descriptors. Adding asymmetric entropy-derived descriptors to models with pRR30 or pRR3.25 slightly improved their AUC or DOR, but at the cost of increasing FNs.

The impact of false positive and false negative results can vary depending on the specific condition being tested for and the context in which the test is performed. For example, a false positive result for a serious condition may lead to unnecessary treatment or further testing. Conversely, a false negative result may delay necessary treatment. In AF, a false-negative diagnosis appears to be more harmful than a positive diagnosis. A patient with undiagnosed AF will not receive antithrombotic treatment and will remain at an increased risk of ischaemic stroke. In contrast, a false-positive result leads to further testing and has no immediate negative consequences [23,24].

The proposed diagnostic methodology, utilizing pRR3.25% and pRR30 parameters along with run-based asymmetric entropy descriptors, presents a streamlined and efficient approach for atrial fibrillation detection. One of the significant advantages of this methodology is its ease of implementation: once the model is trained, the input for the model can be selected in a real-time or online fashion. This process is particularly beneficial as it does not require storing the entire recording in memory, making it highly suitable for continuous monitoring applications. Furthermore, the foundation of this methodology on counting statistics not only simplifies the implementation but also reduces the computational burden, making it an efficient approach for real-time data analysis.

The low computational requirements and the ability to process data incrementally mean that, once trained, these models can be seamlessly integrated into low-energy devices. This adaptability makes the methodology particularly suitable for a wide range of applications, including implantable medical devices, mobile-phone-based health monitoring apps, and home health monitors. The integration of this diagnostic tool into such devices can facilitate continuous, real-time monitoring of cardiac activity, offering significant benefits for the early detection and management of atrial fibrillation in diverse settings and promoting widespread access to advanced healthcare diagnostics.

Our study had several limitations. The analysis was based on RR intervals from 1 min ECGs only, potentially missing longer-term cardiac dynamics that contribute to entropy. We cannot extrapolate our findings to ECGs of shorter or longer duration. Our study focused on the analysis of one-minute length segments, reflecting the specific requirements of our research objectives. Given the substantial dataset comprising over 60,000 such segments, we opted for a stringent criterion to ensure the high quality and reliability of our study outcomes. The decision to discard segments with less than 58 s of total RR interval length aimed to maintain consistency and rigor in our analysis. Furthermore, we would like to reference a relevant study, Buś et al. [25], which investigated the diagnostic value of segment length for AF detection. This study demonstrated that the diagnostic performance of HRV for AF detection is closely associated with the length of the ECG recording. Specifically, the diagnostic properties of HRV indices, particularly pRR50, exhibited significant improvement with longer recordings. For instance, the study reported an area under the curve (AUC) ranging from 0.881 for 5 s segments to 0.980 for 300 s segments, with corresponding diagnostic odds ratios indicating improved performance with longer segment lengths. These findings underscore the importance of segment length in optimizing the diagnostic accuracy of HRV analysis, with longer recordings generally associated with better performance. The sampling frequency of our data was only 128 Hz, which increases the rate of neutral RR intervals for technical rather than physiological reasons. In this study, we tested the ability to detect AF using classic simple logistic regression models. Although we achieved a high accuracy rate of 0.96, there is potential for even higher accuracy rates using more complex machine learning models. ML algorithms may perform better with random forest than with logistic regression. Whether this applies to our results is uncertain. It is noteworthy that the AUC for AF detection by pRR3.25% is already close to 1 and there is not much room for improvement. However, it may be worth exploring a wider range of machine learning models, such as XGBoost [26] or Random Forest [27], to determine their ability to detect AF from the asymmetric entropy of neutral runs. These models may be able to capture more complex relationships between the features and the target variable.

The novelty of our study lies in the comprehensive investigation of AF detection using a novel combination of runs-based asymmetric entropy and pRR30 with pRR3.25% and pRR30. Runs-based asymmetric entropy has not previously been tested as a candidate for AF detection. Another novelty was not to remove neutral runs but to include them and analyse their characteristics, which seems to be useful. We recognize that researchers may exclude neutral runs from analysis as they are often treated as artefacts in the recording process, primarily stemming from poor resolution and a lack of contribution to the structural (run-based) metrics. Indeed, if we consider the neutral runs as unresolved acceleration and deceleration runs, their exclusion in sinus rhythm should not change any results. Excluding neutral runs from analysis in this particular study can be problematic for several reasons. Firstly, although they have no effect on short-term HRV, neutral runs contribute significantly to long-term and total HRV. Removing them could be misinterpreted as data manipulation. Secondly, research specifically investigating neutral runs remains scarce, limiting our understanding of their potential influence. In the context of our study focused on AF detection, the inclusion of neutral runs was of particular importance. A less organized rhythm characterizes AF compared to sinus rhythm. This reduced structure translates into a low probability of finding two or more identical RR intervals within the data, making them comparable to random sequences or shuffled heart cycles. By including neutral runs in our analysis, we aimed to capture and account for the less organized nature of AF. Our data showed a significant difference in entropy associated with neutral runs between AF and sinus rhythm. To our knowledge, this finding is the first demonstration of such an association.

## 5. Conclusions

pRR3.25% stands out as a robust and reliable choice for effectively identifying AF episodes, even in comparison with runs-based asymmetrical entropy. While it is observed that more complex models such as pRR3.25%, pRR30 and H exhibit slightly enhanced performance on a general scale, this marginal improvement is often overshadowed by the substantial drawback of a nearly fourfold increase in false negatives. Notably, it is worth emphasizing that every superior model inherently encompasses the pRR3.25% parameter. This underscores the foundational importance of pRR3.25% in contributing to the diagnostic accuracy of any enhanced model. In practical clinical applications, the single-parameter model with pRR3.25% emerges as the optimal choice, delivering a commendable balance between detection precision and false-negative rates.

Our results suggest that HNR may be a helpful parameter for AF detection, and future studies should investigate its potential further.

## Figures and Tables

**Table 1:** Area under the ROC curve (AUC) and optimal cut-off values for the training set. Median values of classification metrics (from the confusion matrix) for AF detection in the test set using single HRV parameters with the optimal cut-off.

Parameter	AUC	Cut-Off for AF	Accuracy	Sensitivity	Specificity	PPV	NPV	DOR
**pRR3.25%**	0.9727	<72.3684%	95.75	99.60	92.25	92.12	99.60	2931.82
**pRR30**	0.9596	<66.8874%	91.00	95.62	86.80	86.82	95.62	143.70
**HNR**	0.9315	<0.1884	84.86	95.45	75.26	77.83	94.79	63.82
**HAR**	0.6951	<0.7546	67.85	86.83	50.59	61.52	80.86	6.76
**HDR**	0.6686	<0.7726	67.68	91.49	46.00	60.66	85.61	9.16
**H**	0.6186	<2.0647	60.03	85.68	36.69	55.18	73.80	3.47

**Table 2:** Comparison of different metrics for AF detection using univariate and multivariate logistic regression models. The three top metrics with the highest AUC are bolded. pRR3.25% stands out as the most powerful individual metric, exhibiting the highest AUC. Notably, it consistently appears in the top-performing metric pairs and triplets, consistently demonstrating superior performance in distinguishing AF from SR.

Feature	AUC	Accuracy	DOR	FP + FN [%]	FP [%]	FN [%]
**pRR3.25%**	**0.972**	**0.9513**	**643.0**	**4.87**	**3.98**	**0.88**
pRR30	0.959	0.9277	233.5	7.23	5.57	1.65
H	0.613	0.6118	2.6	38.82	24.3	14.52
HAR	0.7	0.6536	3.6	34.64	16.11	18.53
HDR	0.67	0.6155	2.6	38.45	16.91	21.54
HNR	0.93	0.8842	84.5	11.58	9.03	2.55
**pRR3.25 & pRR30%**	**0.978**	**0.955**	**687.1**	**4.5**	**3.57**	**0.93**
pRR3.25% & H	0.986	0.9566	705.6	4.34	3.38	0.96
pRR3.25% & HAR	0.982	0.9549	693.0	4.51	3.59	0.92
pRR3.25% & HDR	0.977	0.9517	623.3	4.83	3.9	0.93
pRR3.25% & HNR	0.974	0.9516	632.3	4.84	3.93	0.91
pRR30 & H	0.973	0.9327	252.3	6.73	5.01	1.72
pRR30 & HAR	0.969	0.9312	240.5	6.88	5.13	1.76
pRR30 & HDR	0.963	0.9279	235.8	7.21	5.57	1.64
pRR30 & HNR	0.96	0.9284	232.9	7.16	5.47	1.69
**pRR30 & pRR3.25%, & H**	**0.988**	**0.9577**	**695.5**	**4.23**	**3.2**	**01.03**
pRR30 & pRR3.25%, & HAR	0.985	0.9581	732.4	4.19	3.22	0.97
pRR30 & pRR3.25%, & HDR	0.982	0.9541	633.7	4.59	3.58	1
pRR30 & pRR3.25%, & HNR	0.979	0.9537	631.3	4.63	3.65	0.99
HNR & HAR, & HDR	0.935	0.8838	82.8	11.62	9.01	2.61

## Data Availability

The data presented in this study are openly available in The Long-Term AF Database at https://doi.org//10.13026/C2QG6Q.

## References

[1] Hindricks G., Potpara T., Dagres N., Arbelo E., Bax J., Blomström-Lundqvist C., Boriani G., Castella M., Dan G., Dilaveris P. (2021). 2020 ESC Guidelines for the diagnosis and management of atrial fibrillation developed in collaboration with the European Association for Cardio-Thoracic Surgery (EACTS): The Task Force for the diagnosis and management of atrial fibrillation of the Europea. Eur. Heart J..

[2] Ble M., Benito B., Cuadrado-Godia E., Pérez-Fernández S., Gómez M., Mas-Stachurska A., Tizón-Marcos H., Molina L., Martí-Almor J., Cladellas M. (2021). Left Atrium Assessment by Speckle Tracking Echocardiography in Cryptogenic Stroke: Seeking Silent Atrial Fibrillation. J. Clin. Med..

[3] Roten L., Goulouti E., Lam A., Elchinova E., Nozica N., Spirito A., Wittmer S., Branca M., Servatius H., Noti F. (2021). Age and Sex Specific Prevalence of Clinical and Screen-Detected Atrial Fibrillation in Hospitalized Patients. J. Clin. Med..

[4] Turagam M.K., Flaker G.C., Velagapudi P., Vadali S., A Alpert M. (2015). Atrial Fibrillation In Athletes: Pathophysiology, Clinical Presentation, Evaluation and Management. J. Atr. Fibrillation.

[5] Lin A.L., Nah G., Tang J.J., Vittinghoff E., A Dewland T., Marcus G.M. (2022). Cannabis, cocaine, methamphetamine, and opiates increase the risk of incident atrial fibrillation. Eur. Heart J..

[6] Rizwan A., Zoha A., Ben Mabrouk I., Sabbour H.M., Al-Sumaiti A.S., Alomainy A., Imran M.A., Abbasi Q.H. (2020). A Review on the State of the Art in Atrial Fibrillation Detection Enabled by Machine Learning. IEEE Rev. Biomed. Eng..

[7] Khan A.A., Lip G.Y.H., Shantsila A. (2019). Heart rate variability in atrial fibrillation: The balance between sympathetic and parasympathetic nervous system. Eur. J. Clin. Investig..

[8] Buś S., Jędrzejewski K., Guzik P. (2022). Statistical and Diagnostic Properties of pRRx Parameters in Atrial Fibrillation Detection. J. Clin. Med..

[9] Piskorski J., Guzik P. (2011). The structure of heart rate asymmetry: Deceleration and acceleration runs. Physiol. Meas..

[10] Goldberger A.L., Amaral L.A.N., Glass L., Hausdorff J.M., Ivanov P.C., Mark R.G., Mietus J.E., Moody G.B., Peng C.-K., Stanley H.E. (2000). PhysioBank, PhysioToolkit, and PhysioNet: Components of a new research resource for complex physiologic signals. Circulation.

[11] Petrutiu S., Sahakian A.V., Swiryn S. (2007). Abrupt changes in fibrillatory wave characteristics at the termination of paroxysmal atrial fibrillation in humans. Europace.

[12] Shannon C.E. (1948). A Mathematical Theory of Communication. Bell Syst. Tech. J..

[13] Levene H., Wolfowitz J. (1944). The covariance matrix of runs up and down. Ann. Math. Stat..

[14] Shapiro S.S., Wilk M.B. (1965). An Analysis of Variance Test for Normality (Complete Samples). Biometrika..

[15] Fawcett T. (2006). An Introduction to ROC analysis. Pattern Recogn. Lett..

[16] Youden W. (1950). Index for rating diagnostic tests. Cancer.

[17] Glas A.S., Lijmer J.G., Prins M.H., Bonsel G.J., Bossuyt P.M.M. (2003). The diagnostic odds ratio: A single indicator of test performance. J. Clin. Epidemiol..

[18] Hastie T., Tibshirani R., Friedman J. (2009). Model Assessment and Selection. The Elements of Statistical Learning.

[19] Richman J.S., Moorman J.R. (2000). Physiological time-series analysis using approximate entropy and sample entropy. Am. J. Physiol. Heart Circ. Physiol..

[20] Liu C., Oster J., Reinertsen E., Li Q., Zhao L., Nemati S., Clifford G.D. (2018). A comparison of entropy approaches for AF discrimination. Physiol. Meas..

[21] Zhao L., Liu C., Wei S., Shen Q., Zhou F., Li J. (2018). A New Entropy-Based Atrial Fibrillation Detection Method for Scanning Wearable ECG Recordings. Entropy.

[22] Żurek S., Grabowski W., Wojtiuk K., Szewczak D., Guzik P., Piskorski J. (2020). Relative Consistency of Sample Entropy Is Not Preserved in MIX Processes. Entropy.

[23] Renshaw A.A., Gould E.W. (2013). Reducing false-negative and false-positive diagnoses in anatomic pathology consultation material. Arch. Pathol. Lab. Med..

[24] Klinkman M.S., Coyne J.C., Gallo S., Schwenk T.L. (1998). False positives, false negatives, and the validity of the diagnosis of major depression in primary care. Arch. Fam. Med..

[25] Buś S., Jędrzejewski K., Guzik P. Impact of Electrocardiogram Length on Diagnostic Properties of Heart Rate Variability Indices in Atrial Fibrillation Detection. Proceedings of the 2022 12th Conference of the European Study Group on Cardiovascular Oscillations (ESGCO).

[26] Chen T., Guestrin C. (2016). XGBoost: A Scalable Tree Boosting System. Proceedings of the 22nd ACM SIGKDD International Conference on Knowledge Discovery and Data Mining.

[27] Breiman L. (2001). Random Forests. Mach. Learn..

